# Paraduodenal Pancreatitis: A Deceptive Abdominal Mass with Unique Histologic Findings

**DOI:** 10.1155/2020/5021578

**Published:** 2020-02-06

**Authors:** Jonathan Zadeh, Anthony Andreoni, Christopher Febres-Aldana, Kritika Krishnamurthy, Jyotsna Kochiyil, Cristina Vincentelli, Kfir Ben-David

**Affiliations:** ^1^Department of Surgery, Mount Sinai Medical Center, Miami Beach, FL, USA; ^2^Department of Pathology, Mount Sinai Medical Center, Miami Beach, FL, USA; ^3^Department of Radiology, Mount Sinai Medical Center, Miami Beach, FL, USA; ^4^Herbert Wertheim College of Medicine, Florida International University, Miami, FL, USA

## Abstract

Paraduodenal pancreatitis (PP) is an uncommon abdominal pathology characterized by scarring of the pancreaticoduodenal space. Diagnosis of this inflammatory process is challenging as its clinical presentation is similar to that of pancreatic cancer. Currently, no definitive radiologic or pathologic features have been established to permit diagnosis of PP without surgical resection. However, the presence of eosinophilic concretions has been reported with increasing frequency in the histologic evaluation of PP. To the best of our knowledge, these concretions are distinctive for PP and not reported in neoplasms commonly involving the pancreaticoduodenal space. Herein, we discuss the case of a 60-year-old man who was found to have PP after pancreaticoduodenectomy for a paraduodenal mass with an initially nondiagnostic biopsy. Retrospective review of the preoperative FNA samples revealed eosinophilic concretions like those found in the final surgical specimen. If the identification of eosinophilic concretions in a background of inflammatory changes was to be accepted as a diagnostic criterion for PP, patients such as ours could be spared the morbidity associated with surgical resection.

## 1. Introduction

Paraduodenal pancreatitis (PP) is a rare chronic inflammatory process involving the pancreaticoduodenal space. The presenting symptoms of PP are often similar to those of pancreatic malignancies [1]. While pancreaticoduodenectomy is occasionally indicated for the treatment of PP, this surgery is often performed solely for diagnostic purposes. This brings significant risk to patients with symptoms that may be treated by less invasive means [2]. Unfortunately, differentiating PP from pancreatic adenocarcinoma and other gastrointestinal cancers prior to resection is challenging. Tissue core biopsies and cytologic preparations of PP-forming lesions are useful to rule out malignancies but have limited value in confirming the diagnosis. Numerous surgical reviews with pathological correlation have been published, but definitive pathologic findings specific for the preoperative diagnosis of PP have not been described [3–6]. While not yet established as a criterion for diagnosis, the presence of eosinophilic concretions has been reported as a unique finding of PP. This histologic finding, which is not characteristic of malignancies in this region, could potentially be used to diagnose PP in biopsies. In appropriate patients, this could avoid the morbidity and mortality associated with formal surgical resection. Herein, we discuss a case of PP found in such a patient who underwent pancreaticoduodenectomy for definitive diagnosis of a duodenal mass identified on imaging studies.

## 2. Case Presentation

The patient discussed herein is a 60-year-old male who presented to the surgical oncology clinic with a one-year history of intermittent abdominal pain. He was previously evaluated by gastroenterology at an outside institution and had undergone a CT scan and an endoscopic ultrasound (EUS). This imaging revealed an approximately 4.5 cm mass between the duodenal bulb and the head of the pancreas with both solid and cystic components. A biopsy was performed during the EUS, but a tissue diagnosis could not be made.

At the time of our initial interview, the patient reported persistence of his abdominal pain as well as 15 pounds of weight loss over the preceding three months. He was otherwise asymptomatic. He admitted to occasional smoking and alcohol use. His family history was negative for GI malignancy. On exam, he was noted to be anicteric and nonjaundiced. Abdominal exam was normal aside from a reducible nontender 3 cm umbilical hernia.

**Figure 1:**
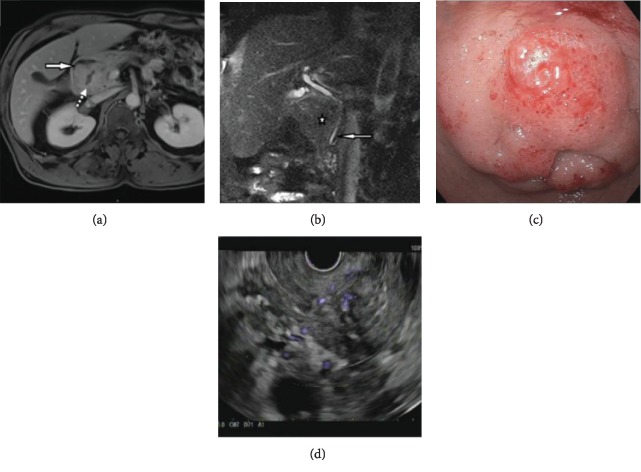
Preoperative endoscopy and imaging. (a) T1 VIBE postcontrast sequence axial MRI acquisition showing a delayed enhancement of the 4.4-centimeter mass along the medial duodenal wall with few internal cystic areas (dashed arrow) and accumulation of fluid in the right anterior paraduodenal space (arrow). (b) T2 HASTE sequence coronal MRI acquisition showing the mass along the duodenum (star) and the distal part of the common bile duct winding around the mass with narrowing at the tip (arrow). (c) Endoscopic appearance of the duodenum showing a polypoid and eroded mucosa. (d) EUS showing an ill-defined heterogeneous paraduodenal lesion causing distortion of the duodenal wall layers.

After the patient's office visit, an MRI was performed for better characterization of the previously identified mass. This showed a 4.4 cm predominantly solid mass in the bulb and second portion of the duodenum with extension into the pancreatoduodenal groove as well as fluid in the right pararenal and paraduodenal spaces (Figures 1(a) and 1(b)). A repeat endoscopy with EUS was performed. Endoscopy showed a nodular, edematous, friable mass in the first portion of the duodenum causing mild stricture of the lumen (Figure 1(c)). EUS revealed a slightly nodular, ill-defined, hypoechoic and heterogeneous submucosal mass in the medial wall of the first portion of the duodenum extending into the duodenal sweep and causing mild stricture. The duodenal wall was thickened with loss of demarcation between layers (Figure 1(d)). Repeat biopsy and fine needle aspiration (FNA) of the mass taken at the time of EUS revealed only duodenal mucosa with dilated lymphatic channels and prominent lymphoid follicles.

Given the remaining diagnostic uncertainty, the patient was discussed in a multidisciplinary fashion at our GI tumor board. It was ultimately decided that the patient should undergo surgical resection for definitive diagnosis. A pancreaticoduodenectomy was performed without intraoperative complication. The patient's postoperative hospitalization was prolonged due to the ileus which was managed with NGT decompression. This appeared to resolve by postoperative day 8 at which point he was discharged. The patient was readmitted two weeks later for severe hiccups and vomiting. He was treated for gastroparesis with erythromycin and Reglan and discharged on hospital day 4. On postoperative follow-up at one and six weeks, the patient reported tolerance of regular diet and freedom from hiccups and nausea.

**Figure 2:**
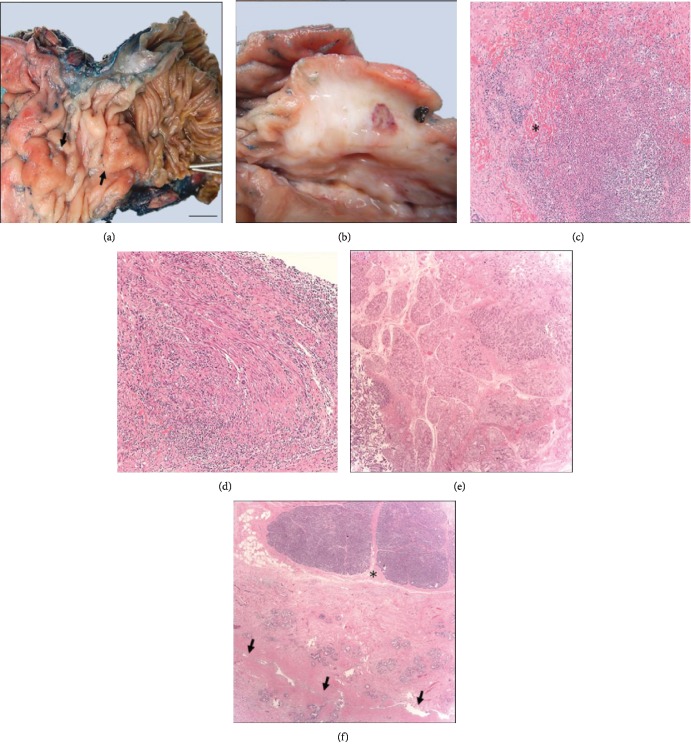
Gross and microscopic examination of the pancreaticoduodenectomy specimen. (a) Thickening of duodenal mucosa with a polypoid appearance in the region of the accessory ampulla (on the left, arrows) and sparing of distal segments (on the right, metallic probes in the ampulla of Vater); bar: 1 cm. (b) Cut section of polypoid areas showing submucosal fibrosis and loss of demarcation of muscularis propria. On the right, a hemorrhagic area corresponding to prior FNA procedure. (c) Microabscess with deposition of keloid-type collagen fibers (asterisk) (H&E, 100x). (d) Dense fibrosis and chronic inflammation, H&E, 200X. (e) Brunner gland hyperplasia (H&E, 100x). (f) Diffuse periductal fibrosis of the accessory duct (arrows) and sparing of adjacent pancreatic parenchyma (asterisk) (H&E, 50x). Additionally identified, prominent lymphoid follicles and peripheral nerve hypertrophy (not shown).

**Figure 3:**
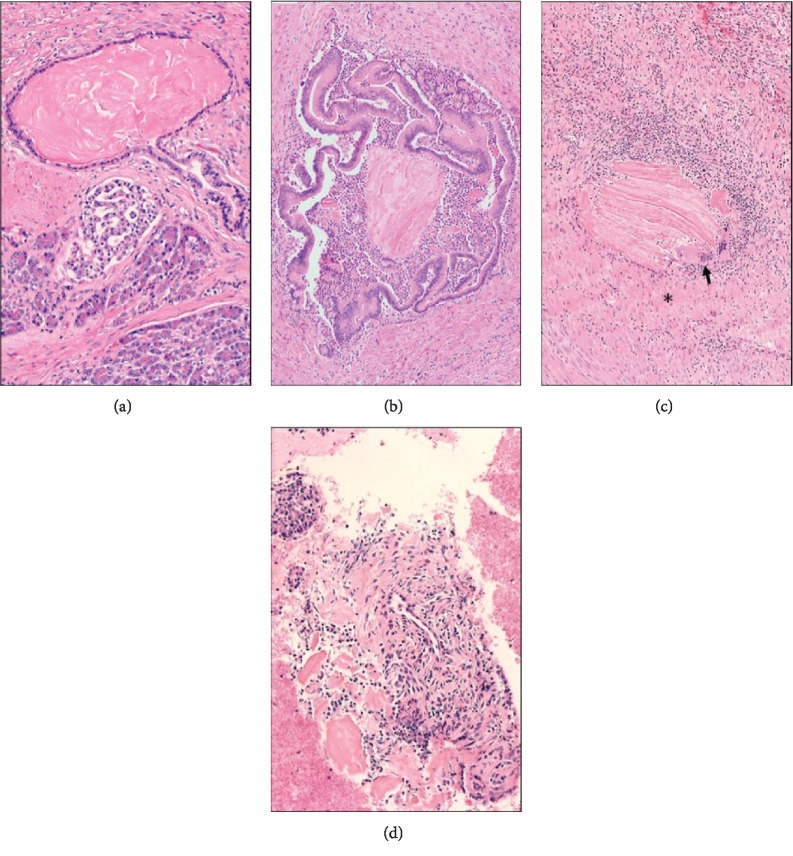
Characteristic eosinophilic concretions in paraduodenal pancreatitis. (a) Eosinophilic concretion within a branch of the pancreatic duct tree enclosed by an attenuated flat epithelium, normal exo- and endocrine pancreas on the bottom (H&E, 400x). (b) Eosinophilic concretion with associated chronic inflammation and dilation of a pancreatic duct branch (H&E, 200x). (c) Leaked concretions leading to a foreign-body giant cell reaction (arrow) with fibrosis (asterisk) (H&E, 200x). (d) Few eosinophilic concretions accompanied by inflammatory cells and spindled stromal cells identified in the cell block of a preoperative FNA aspirate (H&E, 400x).

Gross and microscopic pathologic findings of the surgical sample were suggestive of PP (Figures 2 and 3). The mass effect in this case was secondary to widespread fibroblastic proliferation and local edema (Figure 2(d)). Immunohistochemical stains were negative for beta-catenin, anaplastic lymphoma kinase- (ALK-) 1, and CD-117, which ruled out fibromatosis, inflammatory myofibroblastic tumor, and gastrointestinal stromal tumor, respectively. There was no elevation of serum IgG4 or significant amounts of IgG4-positive plasma cells in the tissue, ruling out autoimmune pancreatitis and IgG4 disease.

## 3. Discussion

### 3.1. Definition

Paraduodenal pancreatitis is a chronic inflammatory process characterized by fibrotic change within the pancreatoduodenal groove. This is an anatomic space bordered by the C-loop of the duodenum, the head of the pancreas, and the common bile duct [6–8]. This lesion was first described in 1970 by Potet and Duclert as cystic dystrophy of heterotopic pancreatic tissue in the duodenal wall. Becker later described it in 1973 as “Rinnenpankreatitis” [9, 10]. Stolte et al. further studied this pathologic process and coined the name “groove pancreatitis” in 1982. In 2004, Adsay and Zamboni described this disease as “paraduodenal pancreatitis,” which was deemed an appropriate unifying term as the inflammatory process predominantly involves the duodenal wall [11].

### 3.2. Incidence

The reported incidence of PP varies significantly in associated surgical reviews of pancreaticoduodenectomy specimens. In a series of 123 surgical Whipple specimens removed for chronic pancreatitis, Stolte et al. identified 30 cases of PP (24.5%) [7]. Becker and Mischke examined 117 Whipple specimens resected for a clinical diagnosis of chronic pancreatitis and identified some degree of groove involvement in 19.5% of the cases with 8.5% of the cases showing groove involvement only [8]. In a more recent case series reviewing the pathology for 882 pancreaticoduodenectomy specimens, only 58 cases (6.6%) met criteria for PP [12]. Similarly, Manzelli et al. identified just 5 cases of PP (3.1%) in their study of 160 pancreaticoduodenectomies performed for chronic pancreatitis [4].

### 3.3. Pathophysiology

The pathogenesis of PP is not well understood. It has been suggested that it may be secondary to chronic inflammation of heterotopic pancreatic tissue in the minor papilla triggered by alcohol use [11]. Involvement of the minor papilla (the orifice to the accessory duct of Santorini) has been implicated in PP as the accessory duct drains pancreatic tissue adjacent to the pancreatic groove. Obstruction of the minor papilla produces increased intraductal pressure and results in leakage of pancreatic fluid into the groove. This triggers an inflammatory response and subsequent stricture of surrounding structures [3]. That process may have occurred in our patient as his surgical specimen was noted to have diffuse fibrosis of the duodenal wall around the minor papilla along with periductal fibrosis and obliteration of the accessory duct (Figure 2(f)).

### 3.4. Risk Factors

Risk factors for PP include alcohol use, peptic ulcer disease, gastric and duodenal resection, pancreas divisum, and duodenal wall cysts [4]. Many of these conditions lead to retrograde flow from the duct of Wirsung into the accessory duct as is seen in obstructive choledocholithiasis [3]. Alcohol use increases the viscosity of pancreatic secretions, which facilitates stagnation of flow and progressive scarring of the ducts. Alcohol consumption also enhances cholinergic tone to the glands of the minor papilla causing Brunner gland hyperplasia [5, 11].

### 3.5. Diagnosis

The diagnosis of PP requires a high degree of clinical suspicion. Patients with PP are frequently middle-aged men with a history of alcohol use or smoking. They generally have an insidious onset of vague gastrointestinal symptoms. The most common presenting symptoms are postprandial abdominal pain (92%), weight loss (78%), and recurrent vomiting (31%) [2]. These symptoms are exacerbated by duodenal stenosis secondary to mass effect. Jaundice may occur late in the disease process if there is stenosis of the common bile duct [5].

While a definitive diagnosis of PP cannot be made on imaging alone, some radiologic features are suggestive of this disease process. On ultrasound, there is thickening of the medial wall of the duodenum with cystic spaces and a sheet-like hypoechoic area in the groove. CT scan with IV contrast will show hypoattenuation with a delayed enhancement of the pancreaticoduodenal groove, fat stranding in the adjacent tissues, and accumulation of fluid in the right anterior paraduodenal and pararenal spaces. MRI with contrast will show delayed or partial enhancement of the soft tissue occupying the groove, appearing hypointense on T1WI (weighted image) and hyperintense on T2WI relative to the pancreas [6]. A hypovascular band between the duodenum and the pancreas referred to as the “sandwich” sign is typical of groove-centered lesions while a “rice ball” pattern is characteristic of lesions involving the head of the pancreas [3]. A lack of demarcation from surrounding structures and fluid accumulation may also indicate PP. Unfortunately, these imaging findings are nonspecific. Acute pancreatitis produces inflammatory changes and fluid collections that evolve rapidly and are also hyperintense on T2WI [12]. Similarly, malignancies such as neuroendocrine tumors and adenocarcinomas are frequently hyperintense on T2WI and demonstrate increased vascularity in contrast imaging with early vivid enhancement [13].

Tissue biopsy must be performed in cases of suspected PP to assess for a neoplastic process. Definitive diagnosis is complicated by the fact that the mucosal and submucosal changes are often subtle and nonspecific. The presence of increased numbers of Brunner glands, spindled stromal cells, and prominent nerve bundles may be used to diagnose PP in cytology specimens, especially if repeat FNAs and biopsies are negative for malignancy [14]. However, these findings are often difficult to identify on cytology and may not be reproducible. Another histological finding commonly seen in PP and observed in this case (Figure 3) is the presence of eosinophilic concretions [3]. These concretions are believed to be the product of proteinaceous secretions that become trapped in and plug pancreatic ducts. This ductal obstruction leads to dilation and wall rupture with extravasation of pancreatic fluid into the surrounding stroma. This event stimulates an inflammatory response with the formation of giant cells, which can also be rich in eosinophils. To the best of our knowledge, similar eosinophilic concretions have not been reported in neoplasms involving the paraduodenal space. Over the last 5 years, our pathology department has evaluated approximately 80 pancreatic specimens determined to contain a neoplasm (both pancreaticoduodenectomy and distal pancreatic resections). 50-60 FNAs of pancreatic neoplasms per year were also examined. None of the specimens had those findings suggestive of paraduodenal pancreatitis. While eosinophilic concretions may be unique to PP, their diagnostic value could be limited due to low sensitivity in FNA preparations. Retrospectively, we were able to identify such concretions on the FNA tissue blocks but not in other cytological preparations like Pap smears and Giemsa stains, suggesting that some histochemical methods may dissolve them (Figure 3(d)). In a previously published series of 3 PP cases, the initial cytological assessment failed to identify any amorphous eosinophilic material. This feature was instead recognized in the histopathology of subsequent excisions [14].

### 3.6. Treatment

In cases where it is felt that PP may be diagnosed without surgical resection, treatment is dependent upon the severity of symptoms. For some patients, even if pancreatic adenocarcinoma is not suspected, pancreaticoduodenectomy may still be appropriate. This is true for individuals suffering from advanced sequela such as intractable pain or severe duodenal stenosis [15]. However, for patients with less severe symptoms, a variety of less invasive therapies have been found to be effective. These include lifestyle modification, somatostatin analogues, pancreatic duct stenting, biliary duct stenting, and duodenal dilation [2].

## 4. Conclusion

With a clinical presentation and radiographic characteristics similar to those of pancreatic and periampullary cancers, paraduodenal pancreatitis possesses a unique diagnostic challenge. Definitive diagnosis often requires formal surgical resection which exposes patients to a relatively high risk of perioperative morbidity for a disease that might otherwise be managed through less invasive means. Surgeons should be aware of this inflammatory process and should discuss the possibility of PP with their pathologists when evaluating patients with a pancreatic groove mass for pancreaticoduodenectomy. In conjunction with imaging findings suggestive of paraduodenal inflammation, the identification of eosinophilic concretions in a background of inflammatory changes may be adequate criteria to confirm PP. While physicians might not presently be willing to diagnose this disease based on such FNA findings, further exploration of those pathologic features may lead to a new standard for PP diagnosis that avoids unnecessary surgical procedures in the future.
